# cFUT8 promotes liver cancer progression by miR-548c/*FUT8* axis

**DOI:** 10.1038/s41392-020-00393-3

**Published:** 2021-01-27

**Authors:** Chong Li, Zhuoyuan Xin, Luyun He, Jing Ning, Kaisu Lin, Jiahui Pan, Jagannatha Rao, Guoqing Wang, Hong Zhu

**Affiliations:** 1 Key Laboratory of Zoonosis Research, Ministry of Education, College of Basic Medical Science, Jilin University, 130021 Changchun, China; 2 Institute of Biophysics, Chinese Academy of Sciences, 100101 Beijing, China; 3 Department of Oncology, The First Affiliated Hospital of Soochow University, 215006 Suzhou, China; 4 Instituto de Investigaciones Científicas y Servicios de Alta Tecnología, Asociación de Interés Público (INDICASAT‐AIP), Panama City, Republic of Panama

**Keywords:** Cancer, Molecular biology

**Dear Editor**,

Hepatocellular carcinoma (HCC) is a serious and lethal malignancy. According to the global cancer statistics of 2018 (GLOBOCAN 2018) provided by the World Health Organization, HCC led to an estimated 781,631 deaths in 2018, comprising 8.2% of the global cancer mortality burden.^1^ Despite improvements in the HCC survival rate, most patients still suffer from metastases and recurrence within 5 years post-surgery.^2^ And it is the unclear molecular pathogenesis that hampers the control of HCC. The discovery of circular RNAs (circRNAs) has the potential to provide new opportunities but also presents several new challenges.^3^ Therefore, there is an urgent need to identify the regulatory role of circRNAs in HCC progression. As a novel molecular regulator, circRNAs have been confirmed to control the progression of HCC, typically acting as RNA sponges.^4,5^ Likewise, in this study, we determined that the circRNA cFUT8 can promote HCC development by binding free miR-548c and inhibiting the miR-548c/*FUT8* regulatory axis.

Firstly, we observed that cFUT8 was overexpressed in HCC tissue, through bioinformatics and qRT-PCR analysis (Fig. 1a and Supplementary Fig. S1a–S1c). The location of cFUT8 was identified using RNAscope (Fig. 1b). Using the Kaplan–Meier survival curve analysis, we determined that cFUT8 expression was negatively associated with the prognosis of patients with HCC (Fig. 1c). Meanwhile, relative cFUT8 expression was also correlated with the pathologic features of HCC, including the BCLC stage and lymphatic invasion (Supplementary Table S1).

**Fig. 1:**
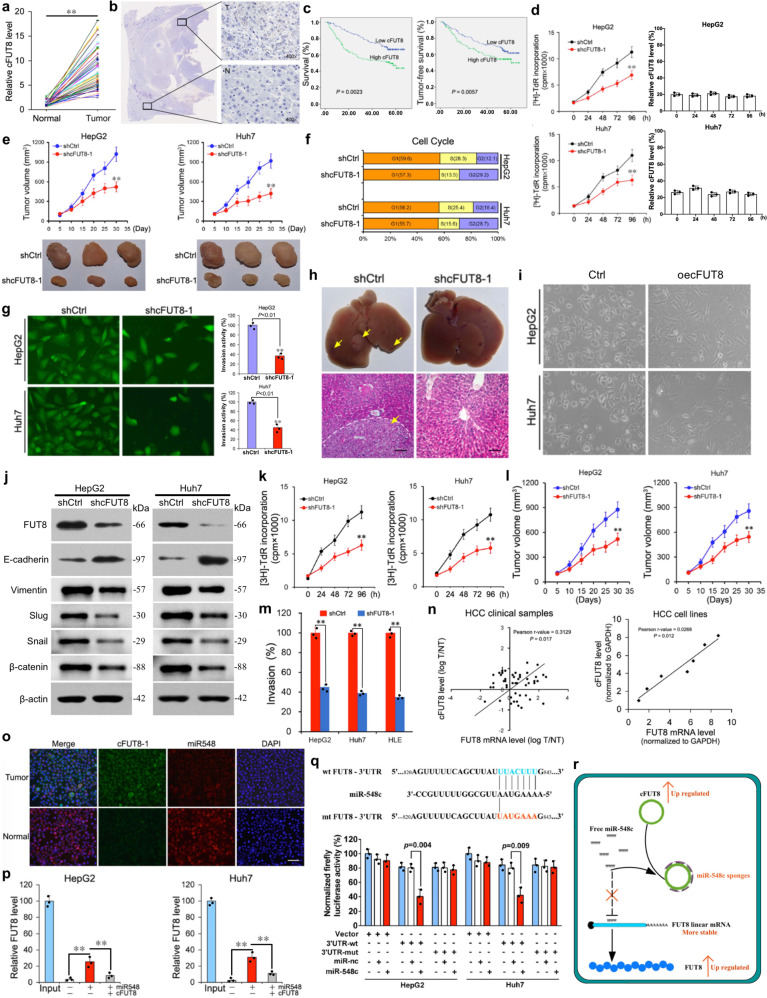
cFUT8 promotes liver cancer progression via miR-548c/FUT8 regulatory axis. **a** Relative cFUT8 expression in liver cancer and para-carcinoma tissues was detected by qRT-PCR (*n* = 57, **p < 0.01). **b** Specimens from patients with HCC show high cFUT8 expression in the cytoplasm of tumor lesions, examined by RNAScope (T represents cancer tissues; N represents para-carcinoma tissues). **c** Utilizing the Kaplan–Meier survival curves analysis, cFUT8 expression was determined to be negatively correlated with overall survival rate and disease-free survival rate of HCC patients. **d** Cell proliferation of HepG2 cells and Huh7 cells with or without cFUT8 knockdown was measured by the CCK-8 assay at different time points (left). Meanwhile, the cFUT8 expression level was examined at each time point using qRT-PCR (right). Mean ± SD of absorbance from each group (*n* = 8) is shown. *p-*values were calculated by paired Student’s *t*-test (**p < 0.01). **e** Knockdown of cFUT8 r**e**markably reduced the volumes of the tumor mass. Volumes of tumors formed by shCtrl or shcFUT8-1 stable clones in NOD-SCID mice were measured using a Vernier caliper on a weekly basis. The top panel shows the mean ± SD (*n* = 5) from each group at each time point. The bottom panel shows the gross view of isolated tumors. *P-*values were calculated by paired Student’s *t*-test (**p < 0.01). **f** Knockdown of cFUT8 in HepG2 and Huh7 led to cell cycle arrest at the G2 stage. **g** Silencing of cFUT8 in HepG2 and Huh7 cells significantly suppressed their invasive ability. The invasive ability of HCC cell lines and their derivatives were determined by Transwell assays (*p < 0.05; **p < 0.01). **h** Injection of cFUT8 stable knockdown clones of Huh7 and HepG2 into the spleen of nude mice. The knockdown of cFUT8 inhibited the metastatic ability of HCC cells in vivo. Representative images of isolated livers (top) and hematoxylin and eosin (H&E) stained liver sections in each group are shown (bottom). **i** Cell morphology of HepG2-oecFUT8 and Huh7-oecFUT8, compared to HepG2-vector and Huh7-vector (blank control), presented with elongated, spindle-like mesenchymal morphology. **j** Silencing cFUT8 expression in HepG2 and Huh7 cells significantly decreased the expression of FUT8 at the protein level. Utilizing western blot, we determined that the expression level of E-cadherin was increased. We also found that other EMT markers, including Vimentin, Slug, Snail, and β-catenin, were significantly suppressed after the silencing of cFUT8 expression. **k** Knockdown of *FUT8* also significantly reduced the proliferation of HepG2 and Huh7 cells. Cell proliferation of HepG2 cells (left) and Huh7 cells (right) with or without *FUT8* knockdown was measured by the CCK-8 assay at different time points (**p < 0.01). **l** Silencing of *FUT8* also remarkably reduced the volumes of the tumor mass. The mean ± SD (*n* = 5) from each group at each time point was shown. All *P-*values were calculated by paired Student’s *t-*test (**p < 0.01). **m** Silencing of *FUT8* also significantly reduced the cell invasion activities of HepG2, Huh7, and HLE cells (**p < 0.01). **n** cFUT8 expression level was positively associated with *FUT8* expression at the mRNA level, both in clinical HCC tissues (left) and HCC cell lines (right). **o** cFUT8 was co-localized with miR-548c in the cytoplasm as shown by FISH. **p** cFUT8 attenuated the inhibitory effects of miR-548c on FUT8 expression, examined by RIP. **q** Dual-Luciferase Reporter Assay System showed that miR-548c could directly bind at 3′UTR of *FUT8*. **r** A putative regulative model of cFUT8 in HCC

In order to identify the regulatory functions of cFUT8 in HCC, we firstly examined the expression levels of cFUT8 in six HCC cell lines (Supplementary Fig. S2a) and constructed cFUT8 stable knockdown cell models using HepG2 and Huh7 cell lines (Supplementary Fig. S2b). We observed that cell proliferation activities were significantly inhibited in vivo and ex vivo (Fig. 1d, e and Supplementary Fig. S2c). However, loss of cFUT8 expression had no detectable influence on the normal liver cell line L02 (Supplementary Fig. S2d), which suggested that cFUT8 may only affect the progression of HCC cells. Using flow cytometric analysis, we detected that the cell cycle was inhibited, with the cycle halting at the G2 stage for most cells (Fig. 1f). Next, we determined that the invasive activities of HCC cells were positively correlated with cFUT8 expression (Fig. 1g; Supplementary Fig. S2a, e). And in this instance, following cFUT8 knockdown, we also observed the metastases formed by cells cannot be detected (Fig. 1h). Following cFUT8 mimic infection, we observed that HepG2 and Huh7 cells with cFUT8-overexpression presented with more elongated, spindle-like mesenchymal morphology (Fig. 1i), which indicated that cFUT8 expression may also be associated with epithelial–mesenchymal transition (EMT). In order to support this inference, we induced EMT via TGF-β treatment among cFUT8-knockdown and control cells and examined the EMT cell markers using western blotting. Following TGF-β induction, we found that E-cadherin expression was upregulated, and vimentin, Slug, Snail, and β-catenin expression was significantly inhibited among cFUT8-knockdown cells (Fig. 1j and Supplementary Fig. S2f). However, the role of cFUT8 during EMT processes still requires further investigation. In addition, using shcFUT8-2, we obtained consistent results (Supplementary Fig. S2g–i), which also verified that cFUT8 promoted the proliferation and invasion activities of HCC. Taken together, these results indicate that cFUT8 could promote HCC cell development, thus maintaining malignant potential.

Based on previous bioinformatics analysis, we hypothesized that the function of cFUT8 in this instance may relate to the binding of free miR-548c and the inhibition of the miR-548c/*FUT8* regulatory axis. To verify this prediction, we examined whether *FUT8* expression levels have any biological relevance in modulating HCC cell activities, which may be related to cFUT8. Firstly, we detected *FUT8* expression levels in liver cancer and normal liver cell lines (Supplementary Fig. S3a). Following knockdown of FUT8 in HepG2 and Huh7 cells (Supplementary Fig. S3b), we observed that cell proliferation, xenograft formation, and cell invasion activities were significantly inhibited (Fig. 1k–m and Supplementary Fig. S3c). Furthermore, stable knockdown of *FUT8* also significantly increased E-cadherin protein levels (Supplementary Fig. S3d), similar to the effects observed in cFUT8-knockdown. Next, we increased the free miR-548c levels within HCC cell lines by infecting with miR-548c mimics. And then, we determined whether free miR-548c levels have any biological relevance to cFUT8 expression. As expected, the regulatory effects of cFUT8 were significantly counteracted by the increased presence of free miR-548c. After adding exogenous miR-548c, the protective effects of cFUT8 on *FUT8* expression were significantly reduced, and cell invasion, migration, and xenograft formation activities were also inhibited (Supplementary Fig. S4a–f).

Finally, to verify the predicted regulatory pattern of cFUT8, via binding of free miR-548c and indirectly increasing FUT8 expression, we used *Pearson* correlation analysis. Thus, we determined that *FUT8* expression was positively correlated with cFUT8 expression at the mRNA level, in both HCC clinical tissue samples and cell lines (Fig. 1n). Then, we detected the subcellular location of both cFUT8 and miR-548c using fluorescence in situ hybridization (FISH). The results showed that cFUT8 and miR-548c were predominantly co-localized in the HCC cell cytoplasm (Fig. 1o). To determine whether endogenous cFUT8 may serve as a binding platform for miR-548c, we conducted RNA immunoprecipitation (RIP) in HepG2 and Huh7 cells. Strikingly, endogenous miR-548c binding to cFUT8 increased more than 30-fold (Supplementary Fig. S5a). Similarly, endogenous cFUT8, pulled down by miR-548c, was also significantly increased at almost 40-fold in these cells (Supplementary Fig. S5b). With similar processing, expression of *FUT8* was significantly enhanced when miR-548c was present (Supplementary Fig. S5c). However, this enrichment was attenuated when coupled with cFUT8 (Fig. 1p). When the free miR-548c level was increased, we identified that FUT8 expression was significantly inhibited at both the mRNA and protein levels (Supplementary Fig. S5d, e). Meanwhile, we performed a dual-luciferase reporter assay to assess whether FUT8 is a direct target of miR-548c. As shown in Fig. 1q, the luciferase activity in both HepG2 and Huh7 cells was significantly reduced by miR-548c mimic transfection, compared with the negative control. Therefore, by binding free miR-548c in the cytoplasm, cFUT8 may indirectly control the expression of *FUT8*.

Overall, we proposed a cFUT8/miR-548c/FUT8 regulatory model for HCC progression (Fig. 1r). During EMT, cFUT8 may dramatically increase the expression levels of *FUT8* by controlling the availability of miR-548c in HCC cancer cells. Our study thus highlights the pathologic role of cFUT8 in HCC, which may aid in the development of novel diagnostic and therapeutic approaches for HCC.

## Supplementary information

Supplementary_Materials
