# Supplementary material for: cFUT8 promotes liver cancer progression by miR-548c/FUT8 axis
Source: Signal Transduct Target Ther. 2021 Jan 27;6:30. doi: 10.1038/s41392-020-00393-3 (PMC7838165; doi:10.1038/s41392-020-00393-3)
Supplement: Supplementary file 1 — Supplementary_Materials [file 41392_2020_393_MOESM1_ESM.pdf]

Supplementary Materials for

cFUT8 promotes liver cancer progression by miR-548c/*FUT8* axis

Chong Li[1,3#], Zhuoyuan Xin[1,#], Luyun He[3,#], Jing Ning[2], Kaisu Lin[2], Jiahui Pan[1], Jagannatha Rao[4], Guoqing Wang[1,*], Hong Zhu[2,*].

[#] These authors contributed equally to this work.

Correspondence to:
Prof. Hong Zhu (zhuhong_jasmine@suda.edu.cn)
Prof. Guoqing Wang (qing@jlu.edu.cn)

This PDF file includes:

Figures. S1 to S5
Tables S1

Figure. S1.

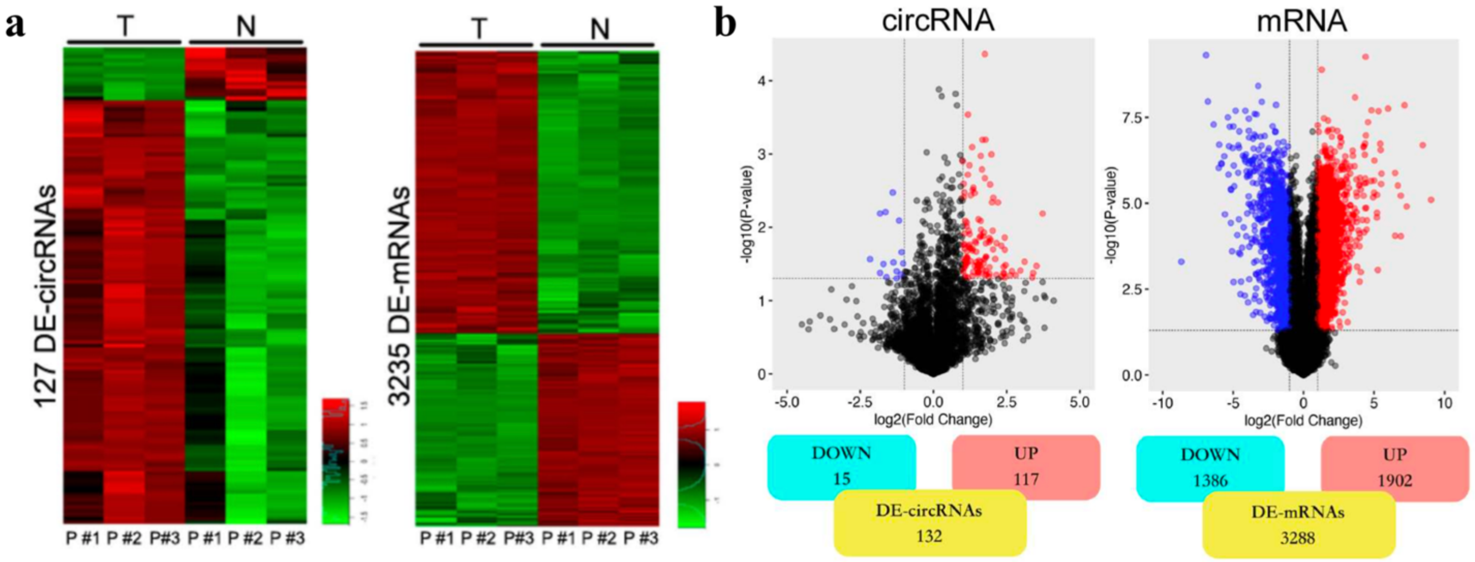

c

cicrRNA	FC (abs)	Regulation	Chrom	Strand	txStart	txEnd	GeneSymbol
hsa_circRNA_101368	11.4439852	up	chr14	+	66028054	66028484	FUT8
hsa_circRNA_102399	10.5805209	up	chr19	+	1032390	1032695	CNN2
hsa_circRNA_103639	10.3668111	up	chr4	+	52729602	52758017	DCUN1D4
hsa_circRNA_103847	8.6475448	up	chr5	+	61745753	61747760	IPO11
hsa_circRNA_101238	7.8155052	up	chr13	+	28748408	28752072	PAN3
hsa_circRNA_100668	6.9817699	up	chr10	−	103552595	103570071	MGEA5
hsa_circRNA_100542	6.3880539	up	chr10	−	7290509	7327916	SFMBT2
hsa_circRNA_100226	6.1438262	up	chr1	−	51868106	51874004	EPS15
hsa_circRNA_100737	5.510219	up	chr11	−	1307231	1317024	TOLLIP
hsa_circRNA_101715	5.4138449	up	chr16	+	14738130	14738466	BFAR

Figure S1. a The Heatmaps of aberrantly expressed circRNAs and mRNAs from three pairs of HCC and adjacent normal tissues, using R version 3.6.0. **b** The Volcano plots of aberrantly expressed circRNAs and mRNAs, using R version 3.6.0. **c** The top 10 significantly up-regulated circRNAs in HCC tissues.

Figure. S2.

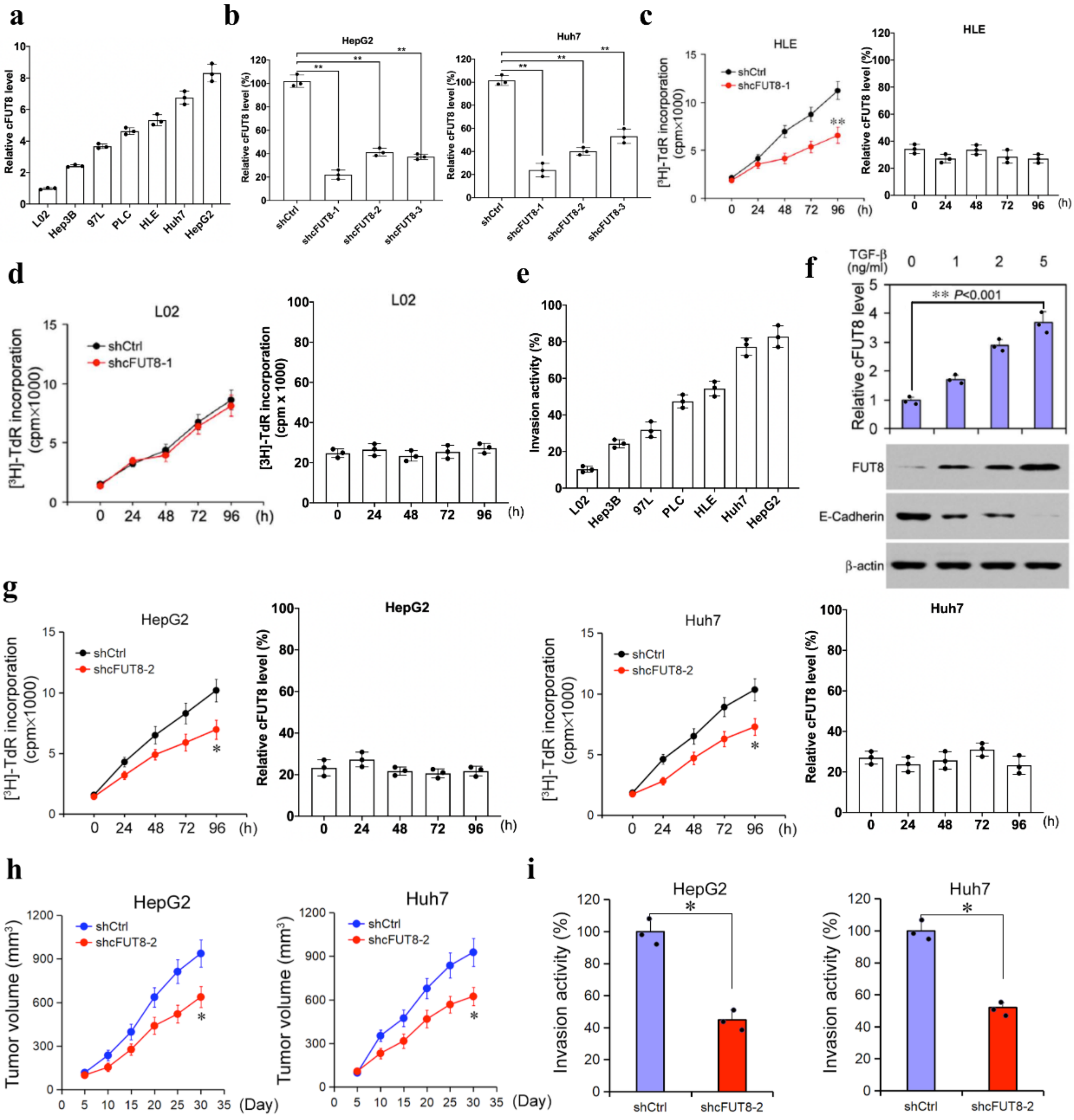

Figure S2. a cFUT8 expression was highly expressed among all six HCC cell lines, utilizing qRT-PCR assay. **b** Knockdown efficiency of three shRNAs against cFUT8 among HepG2 and

Huh7 cell lines was quantified by qRT-PCR (*$p < 0.05$, **$p < 0.01$). **c** Cell proliferation of HLE cells with or without cFUT8 knockdown was measured by the CCK-8 assay at different time points (left). Meanwhile, cFUT8 expression level was examined at each time point using qRT-PCR (right). Mean ± SD of absorbance from each group (n = 8) is shown. *P*-values were calculated by paired Student's *t* test. **d** Knockdown of cFUT8 had no detectable influence on the normal liver cell line L02. **e** Using the Transwell assay, we determined that the expression levels of cFUT8 were positively correlated with the invasive ability of HCC cell lines. **f** *FUT8* and cFUT8 is up-regulated and associated with TGF-β-induced EMT progression. Lanes 1–4 represents short-term exposure to different doses of TGF-β (0–5 ng/mL), respectively. The mean ± SD of relative folds of cFUT8 expression (against untreated) from three independent experiments are shown. *P*-values were calculated by one-way ANOVA. *FUT8* and cFUT8 expression was elevated upon TGF-β treatment in a dose-dependent manner. **g-i:** Using shcFUT8-2 to knock-down cFUT8 among HepG2 and Huh7 cells, we found the cell proliferation activities (g), xenografts formation activities (h) as well as cell invasion activities (i) were significantly inhibited.

Figure. S3.

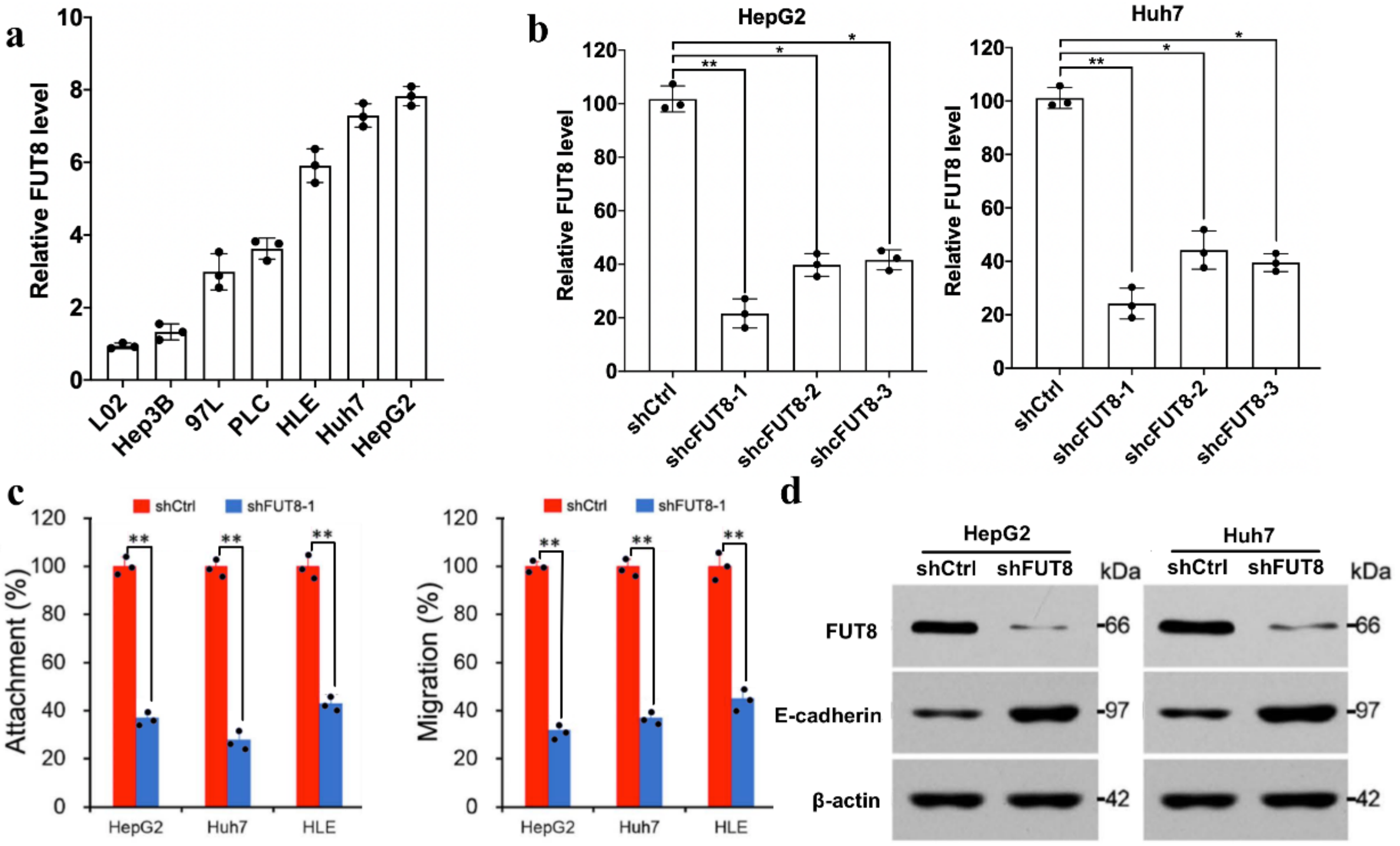

Figure S3. a The *FUT8* expression level was positively correlated with malignancy. **b** Knockdown efficiency of three shRNAs against FUT8 was quantified by qRT-PCR. **c** Using the BCA Method, we examined the relative cell concentration that attached to a Petri plate. The silencing of *FUT8* significantly reduced the intercellular attachment. Cell migration activities were also inhibited via Transwell assay. **d** Knockdown of FUT8 significantly increased the expression of E-cadherin in HepG2 and Huh7 cells, which were detected by western blot.

Figure. S4.

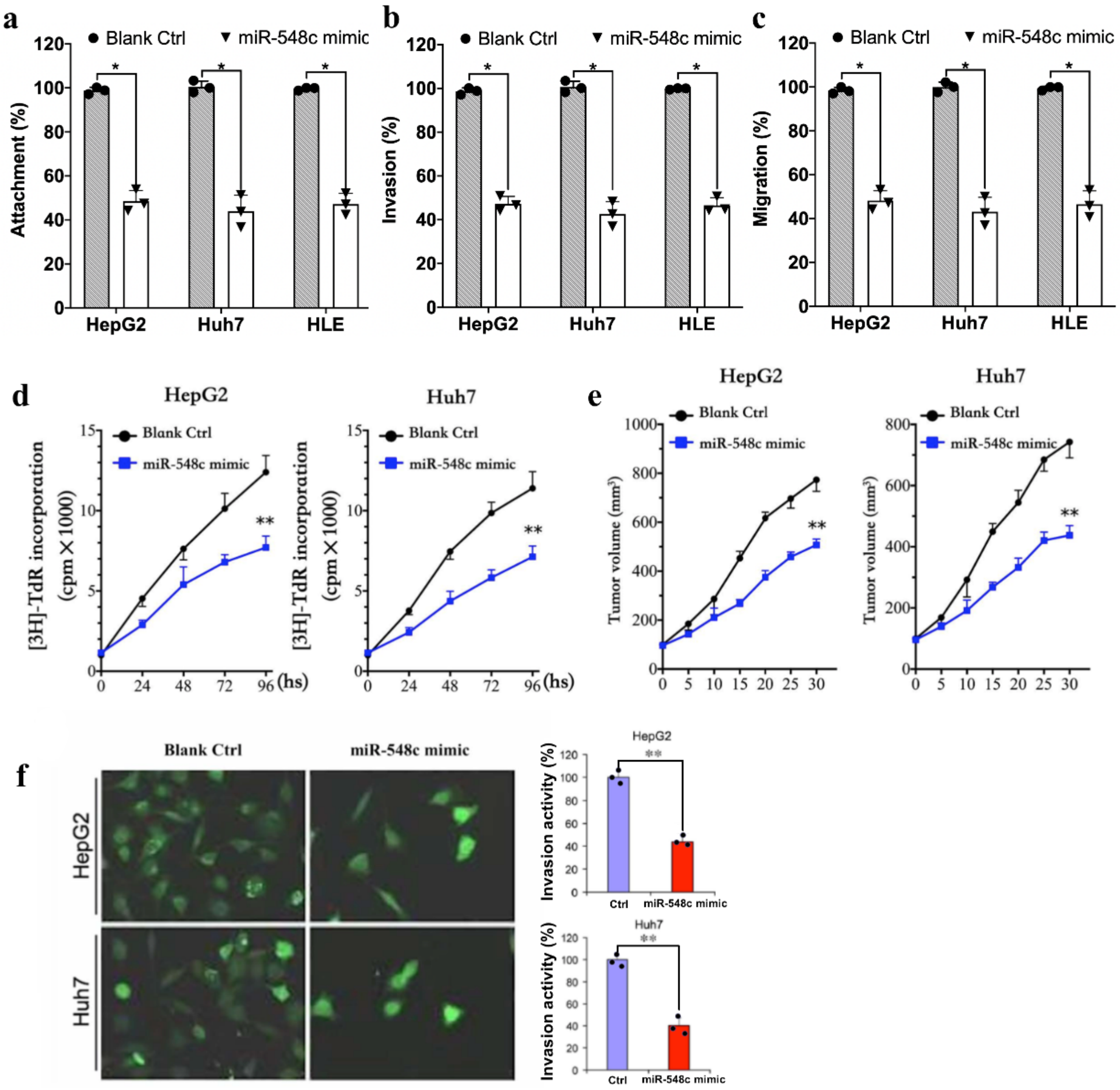

Figure S4. a-c Increasing free miR-548c level significantly reduced the intercellular attachment (a) the cell invasion activities (b), and the cell migration activities (c) of HepG2, Huh7, and HLE cells were also detected. **d-e:** Increasing free miR-548c levels in HepG2 and Huh7 significantly reduced their proliferation rates, both *in vivo* and *ex vivo*. **f** Increasing free miR-548c levels in

HepG2 and Huh7 cells significantly suppressed their invasive ability. The invasive ability of HCC cell lines and their derivatives were determined by Transwell assay.

Figure. S5.

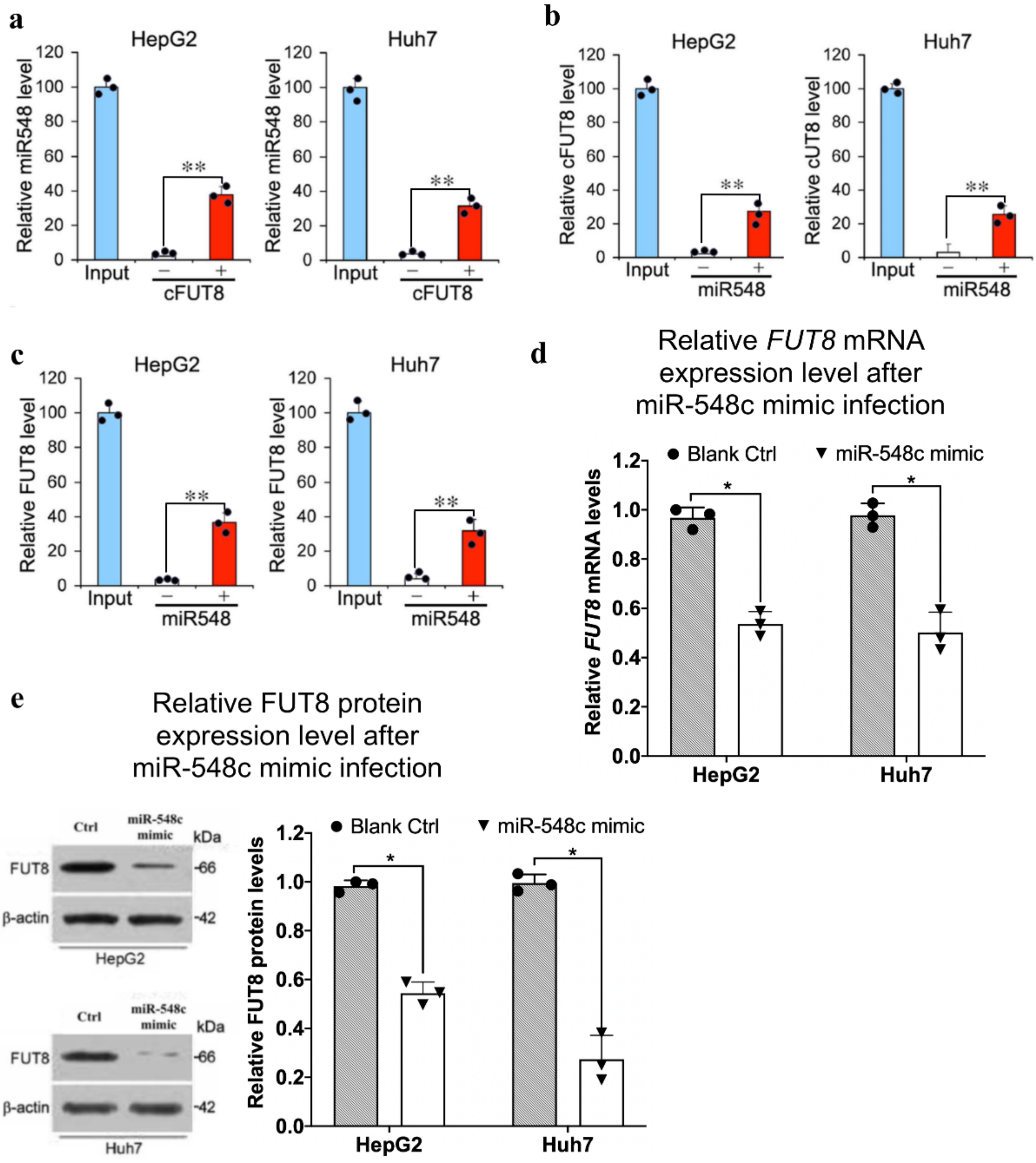

a
HepG2
Relative miR548 level
Input
cFUT8
**
Huh7
b
Relative cFUT8 level
Relative cUT8 level
miR548
c
Relative FUT8 level
d
Relative FUT8 mRNA expression level after miR-548c mimic infection
Blank Ctrl
miR-548c mimic
Relative FUT8 mRNA levels
*
e
Relative FUT8 protein expression level after miR-548c mimic infection
Ctrl
miR-548c mimic
kDa
FUT8
β-actin
66
42
Relative FUT8 protein levels

Figure S5. a Endogenous miR-548c was pulled-down and enriched with a cFUT8-specific probe, and then detected by qPCR. Mean ± SD of absorbance from each group was shown. *P*-values were calculated by paired Student's *t* test. **b** Endogenous cFUT8 in HCC cell lysis was pulled-down and enriched with a miR548-specific probe, and then detected by qPCR. **c** Endogenous *FUT8* was pulled-down and enriched almost 40-fold using a miR-548c-specific probe, and then detected by qPCR. **d-e** Increasing free miR-548c levels significantly suppressed the mRNA (d) and protein level (e) of *FUT8*.

Table S1.

Correlations among cFUT8 expression levels and clinicopathological characteristics in HCC patients (n=217)

**Clinicopathologic parameters**		**Total (n=217)**	**cFUT8 expression#**		***P* value**
			**High**	**Low**	
Age (Years)	≤50	62	34	28	＞0.05
	＞50	155	93	62	
Sex	Male	152	93	59	＞0.05
	Female	65	34	31	
BCLC| stage	A	76	33	43	＜0.001
	B	63	46	17	
	C	67	41	26	
	D	11	7	4	
Differentiation	High or Moderate	130	69	61	＜0.001
	Low	87	58	29	
Number of tumors	Solitary	75	44	31	＞0.05
	Multiple	142	83	59	
Lymphatic invasion	Negative	54	17	37	＜0.005
	Positive	85	65	20	
	Unknown	78	45	33	

The median value was used as the cutoff threshold.
